# Timing and Adherence Matter for Sodium–Glucose Cotransporter‐2 Inhibitors in Heart Failure

**DOI:** 10.1161/JAHA.124.037035

**Published:** 2025-03-21

**Authors:** Mehmet Birhan Yilmaz, Ahmet Celik, Anil Sahin, Tugce Colluoglu, Dilek Ural, Arzu Kanik, Naim Ata, Mustafa Mahir Ulgu, Şuayip Birinci

**Affiliations:** ^1^ Department of Cardiology, Faculty of Medicine Dokuz Eylül University Izmir Turkey; ^2^ Department of Cardiology, Faculty of Medicine Mersin University Mersin Turkey; ^3^ Department of Cardiology, Faculty of Medicine Sivas Cumhuriyet University Sivas Turkey; ^4^ Department of Cardiology, Faculty of Medicine Karabük University Karabük Turkey; ^5^ Department of Cardiology, Faculty of Medicine Koç University Istanbul Turkey; ^6^ Scientific Director of MedicReS, Medical & Clinical Research Support Society, Department of Biostatistics and Medical Informatics, Faculty of Medicine Mersin University Mersin Turkey; ^7^ General Directorate of Information Systems Ministry of Health Ankara Turkey; ^8^ Deputy Minister of Health Ministry of Health Ankara Turkey

**Keywords:** adherence, diabetes, heart failure, SGLT‐2 inhibitors, timing, Heart Failure, Mortality/Survival

## Abstract

**Background:**

It is imperative to maintain the use of sodium–glucose cotransporter‐2 inhibitors (SGLT‐2is) in patients with diabetes both after the index diagnosis of heart failure (HF) and even prior to the index diagnosis of HF. We aimed to investigate whether timing of SGLT‐2 is before the index diagnosis of HF, and second, adherence to SGLT‐2is in the form of the proportion of days covered metric matter in patients with HF and diabetes.

**Methods and Results:**

All‐cause death up to 7 years were evaluated in HF with diabetes from the subgroup analysis of TRends‐HF (TRends in Heart Failure in Türkiye). Patients with HF and diabetes, who were prescribed an SGLT‐2i either before or after the index diagnosis of HF were identified, categorized according to duration of exposure before the index HF diagnosis and according to proportion of days covered after the index diagnosis of HF, and compared with nonusers. There were 1 229 833 patients with HF and diabetes in the cohort. A total of 247 987 were on an SGLT‐2i and had available timing data, and 14.06% had SGLT‐2i on board before the index HF diagnosis. Median duration of SGLT‐2i exposure before the index HF diagnosis was 417 days. Prognosis was the best among patients with diabetes who were prescribed an SGLT‐2i before the index diagnosis of HF with an exposure more than median duration. Of note, among patients who were prescribed an SGLT‐2i after the index HF diagnosis; there was a numerically graded increase in all‐cause mortality rate such that a >10% decrease in SGLT‐2i proportion of days covered was associated with a 59% increase in all‐cause death (hazard ratio, 1.21–2.09).

**Conclusions:**

Regardless of time or adherence, SGLT‐2is offer a remarkable all‐cause death benefit to patients with HF and diabetes. SGLT‐2is' all‐cause death benefit for patients with HF and diabetes was greatest when it was prescribed before the HF index diagnosis. Poor adherence to SGLT‐2is was associated with worsening survival in patients with HF and diabetes following the diagnosis of index HF.

**Nonstandard Abbreviations and Acronyms**
DAPA‐HF: Dapagliflozin and Prevention of Adverse Outcomes in Heart Failure
DECLARE: Dapagliflozin Effect on Cardiovascular Events
EMPAREG‐OUTCOME: Empagliflozin Cardiovascular Outcome Event Trial in Type 2 Diabetes Mellitus Patients
EMPEROR‐REDUCED: Empagliflozin Outcome Trial in Patients With Chronic Heart Failure and a Reduced Ejection Fraction
PDC: proportion of days covered
SGLT‐2i: sodium–glucose cotransporter‐2 inhibitors
TRends‐HF: TRends in Heart Failure in Türkiye

**Clinical Perspective**

**What Is New?**

- Prolonged adherence of sodium–glucose cotransporter‐2 inhibitors (SGLT‐2is) before heart failure (HF) diagnosis may be associated with improved survival in patients with HF and diabetes; notably, a shorter duration of SGLT‐2i use before HF diagnosis may be linked to even higher mortality risk compared with initiation of an SGLT‐2i after HF diagnosis.
- Proportion of days covered <90% for SGLT‐2i use after HF diagnosis may significantly increase the risk of mortality in patients with HF and diabetes.

**What Are the Clinical Implications?**

- Efforts are needed to improve survival in patients with HF and diabetes in terms of implementation of SGLT‐2is, particularly addressing adherence to SGLT‐2is in patients with HF and diabetes.

Sodium–glucose cotransporter 2 inhibitors (SGLT‐2is) have been on the market as part of a therapeutic armamentarium against type 2 diabetes since 2013 along with recent robust evidence of cardiorenal protection, particularly of heart failure (HF) prevention and management beyond diabetes.^1^ Two of these agents, namely, dapagliflozin and empagliflozin, have recently been shown to improve outcomes in patients with symptomatic HF, irrespective of ejection fraction.^2^ In addition, these agents are well established antidiabetics in high‐risk patients and hence strongly indicated in patients with diabetes before the index diagnosis of HF according to recent guidelines.^3^ Of note, medication adherence is an integral part of successful management of chronic noncommunicable diseases. As such, medication nonadherence is a critical problem among patients with HF as well, and it is linked to poor prognosis.^4^, ^5^ Recently, withdrawal of empagliflozin at the end of the HF clinical trials has been shown to yield increased risk,^6^ rendering adherence to these agents critically important for prognosis. However, prognosis related to the timing and adherence to SGLT‐2is has not been well studied before, although adherence and persistence is a matter of ongoing research not only for patients with diabetes but recently for patients with HF.^7^, ^8^

In this real‐world analysis, we aimed to test (1) the timing hypothesis in the form of duration in days of SGLT‐2i use before the index diagnosis of HF matters among patients with diabetes and (2) SGLT‐2i adherence in the form of the proportion of days covered (PDC) metric after index HF diagnosis matters in relation to all‐cause death among patients with HF and diabetes from the nationwide TRends‐HF (TRends in Heart Failure in Türkiye) study.

## Methods

**Figure 1:**
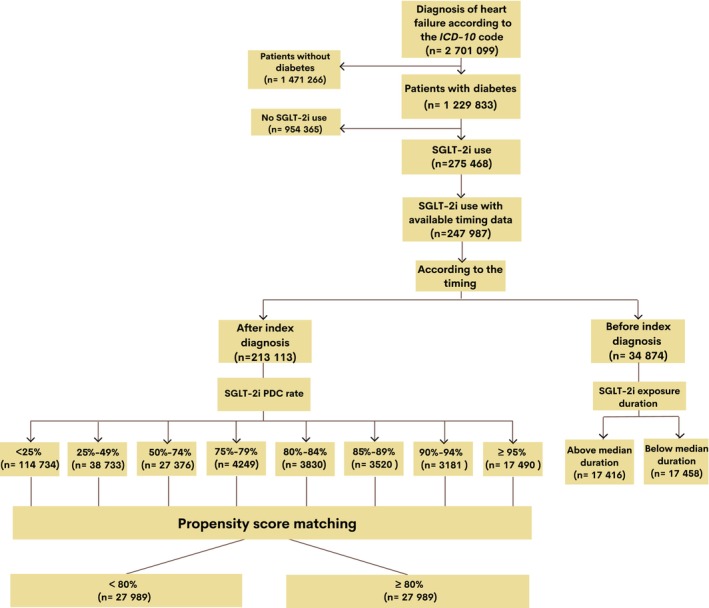
Flowchart of the included cohort for the analyses. *ICD* indicates *International Classification of Diseases*; and SGLT‐2i, sodium–glucose cotransporter‐2 inhibitor.

This is a subgroup analysis (Figure 1) from anonymized data from the Turkish Ministry of Health's National Electronic Database for adult patients with HF, derived from TRends‐HF.^9^ The database consisted of data of 85 million citizens between January 1, 2016, and December 31, 2022. The diagnosis of HF was based on the following *International Classification of Diseases*, *Tenth Revision* (*ICD‐10*) codes: I50.0 (congestive HF), I50.1 (left ventricular dysfunction), I50.9 (HF, unspecified), I11.0 (hypertensive heart disease with congestive HF), I13.0 (hypertensive heart and chronic kidney disease with congestive HF), I13.2 (hypertensive heart and chronic kidney disease with congestive HF and renal failure), and I42.0 (dilated cardiomyopathy). Comprehensive identification of underlying HF pathogeneses and concomitant diseases was achieved by referencing at least 1 *ICD‐10* code recorded in the National Electronic Database up to and including the date of HF diagnosis (Table S1). All included medications in the study were categorized according to the Anatomical Therapeutic Chemical classification system (Table S1) and data regarding initial prescription date, last prescription date (by calculating the last day of available tablets in the prescription), and hence duration of exposure to dapagliflozin or empagliflozin, the only available SGLT‐2is in Turkey, were captured from physician prescription claims. Hence, data regarding the duration of any SGLT‐2i exposure among patients with diabetes before the index diagnosis of HF were captured along with SGLT‐2i adherence in the PDC metric after index diagnosis of HF.

If the SGLT‐2i initial prescription date was before the index date of the HF diagnosis, this was categorized as SGLT‐2i before the index diagnosis of HF (ie, prescribed when the patients with diabetes were either in stage B or stage A HF) and if it was after the index HF diagnosis date, this group was categorized as SGLT‐2i after the index HF diagnosis (ie, stage C HF).

To test timing hypothesis, SGLT‐2i exposure duration in days before the index diagnosis of HF among patients with diabetes was calculated according to physician electronic prescription claims (according to the first and the last prescription day of any dose of either dapagliflozin or empagliflozin). Hence, for those whose initial SGLT‐2i prescription date was before the index HF diagnosis, duration of SGLT‐2i exposure was calculated by subtracting anticipated date of the last dose of SGLT‐2i according to the last prescription (or index date of HF diagnosis if it exceeds that time window) from the initial prescription date of the SGLT‐2i. Median duration was calculated then to yield those above and below median duration.

On the other hand, SGLT‐2i adherence after the HF diagnosis was calculated in the form of PDC metric as shown previously.^10^ Hence, medication adherence was calculated for dapagliflozin or empagliflozin, and patients filling a prescription for dapagliflozin or empagliflozin were considered by the PDC, which is calculated as the number of days in the measurement period covered by physician prescription claims for dapagliflozin or empagliflozin. Poor adherence was defined as a PDC <80%.^11^

Recorded procedures and tests were based on the Health Implementation Declaration codes in the Republic of Turkey Health Implementation Declaration. Number of cardiology outpatient visits per year and emergency department admissions per year were calculated. Total length of stay in the hospital for all‐cause hospitalization was obtained and length of stay in the hospital per year was calculated by dividing by years of follow‐up. Data on death were obtained from the Turkish Death Notification system. Available variables were obtained from the National Electronic Database. As this study was designed as a retrospective observational cohort analysis, the requirement for obtaining informed consent was waived. Furthermore, all necessary ethical approvals and permissions for the study protocol were obtained from the Ministry of Health of the Republic of Turkey. The authors declare that all supporting data are available within the article and its online supplementary files. The study protocol was conducted in accordance with the Declaration of Helsinki and was approved by Ministry of Health of Turkey (Approval No. 95741342–020).

## Statistical Analysis

All count data were subjected to frequency calculations. For categorical variables, cross‐tabs were produced, and values were given as the number of cases and percentages. In both continuous and time‐to‐event data, the median and interquartile range (25%–75%) were used to express the data.

PDC was calculated by framing the date of the first/index prescription for any dose of dapagliflozin or empagliflozin and the last date of the prescription plus the days covered with the last prescription or death. Number of days on dapagliflozin or empagliflozin was divided by the number of days of drug coverage in the PDC framework.

Considering SGLT‐2i users before (those with above‐ and below‐median duration) and after index HF diagnosis versus nonusers, age and sex‐adjusted Cox regression analyses were generated.

After the index diagnosis of HF, SGLT‐2i adherence in the form PDC was further classified into 4 major categories of SGLT‐2i adherence as <25%, 25% to 49%, 50% to 74%, and ≥75%. For precise accuracy, narrower additional adherence subgroups were generated as 75% to 79%, 80% to 84%, 85% to 89%, 90% to 94%, and 95% to 100% PDC for SGLT‐2i. A similar survival analysis was executed according to SGLT‐2i adherence after index HF diagnosis in age‐ and sex‐adjusted Cox regression analysis.

To address potential selection bias and estimate effect of good versus poor adherence in this study, propensity score matching was generated 1:1 to compare those with and without an 80% SGLT‐2i PDC adherence rate after index HF diagnosis. First, a logistic regression model including the clinically relevant variables as confounders was fitted to estimate the propensity scores, representing the probability of good versus poor adherence on observed covariates. The logistic regression model included age, sex, history of myocardial infarction, atrial fibrillation, usage of β blockers, renin–angiotensin–aldosterone system inhibitors, and mineralocorticoid receptor antagonists as confounders. We selected clinically relevant confounders using Cohen's effect size measures both before and after regression analysis. We specifically used Cohen's *d* statistics to evaluate the magnitude of differences in continuous variables and Cohen's *h* statistics to assess the effect size for categorical variables. The definition of effect size, as calculated by the *d* coefficient (*d* = 0.2, small effect size, *d* = 0.5, medium effect size; and *d* = 0.8, large effect size) and *h* coefficient (*h* = 0.1, small difference; *h* = 0.3, medium difference; and *h* = 0.5, large difference), respectively.^12^ Subsequently, a nearest‐neighbor matching algorithm was applied, pairing treated and untreated units with similar or identical propensity scores. The matched data set was then used for subsequent analysis to minimize confounding effects and enhance the comparability of both groups and Cox proportional hazard regression analysis was generated by checking the proportionality assumption. The SPSS 25.0 software program (IBM Corp, Armonk, NY) and E‐PICOS AI (MedicReS, New York, NY) were used for all statistical tests.^13^

## Results

In this analysis, of 2.7 million patients with HF, there were 1.23 million patients with HF and type 2 diabetes, with a median age of 69 years (45.4% men). There were 247 987 patients with HF and diabetes who had available timing data for SGLT‐2i prescriptions and baseline characteristics were represented in the Table and Table S2. Of those, 34 874 (14.06%) patients with HF and diabetes were prescribed an SGLT‐2i before the index HF diagnosis, with a median duration of SGLT‐2i exposure of 417 days, while 213 113 patients with HF and diabetes were prescribed an SGLT‐2i after the index diagnosis of HF (Figure 1).

**Table 1:** Baseline Characteristics of All Patients and Propensity Score Matching Analysis of Patients Using SGLT‐2is After Index HF Diagnosis

Characteristics	All patients on SGLT‐2i (n=247 987)	SGLT‐2i after HF diagnosis*			
		<80% (n=27 989)	≥80% (n=27 989)	*P* value	Cohen for comparison
Age, y, mean±SD	63.5±9.7	62.5±9.5	62.5±9.5	1.000	0
Sex, female, n (%)	126 762 (51.1)	13 953 (49.9)	13 953 (49.9)	1.000	0
Anemia, n (%)	111 111 (44.8)	12 352 (44.2)	12 258 (43.9)	0.423	0.01
Chronic obstructive pulmonary disease, n (%)	105 526 (42.6)	11 839 (42.4)	10 677 (38.2)	<0.001	0.08
History of myocardial infarction, n (%)	66 867 (27.0)	7621 (27.3)	7621 (27.3)	1.000	0
Atrial fibrillation, n (%)	86 771 (35.0)	8471 (30.3)	8471 (30.3)	1.000	0
eGFR, mL/min per 1.73 m^2^	81.6 (63.2–95.4)	82.9 (64.4–96.4)	84.3 (66.0–96.9)	<0.001	0.06
Potassium, mmol/L	4.2 (3.8–4.6)	4.3 (3.9–4.6)	4.2 (3.8–4.6)	0.392	0.03
Uric acid, mg/dL	5.5 (4.4–6.8)	5.5 (4.4–6.8)	5.2 (4.2–6.4)	0.027	0.06
Hemoglobin, g/dL	12.9 (10.0–15.7)	13.0 (10.2–15.7)	13.2 (10.2–16.0)	0.504	0.02
BNP, pg/mL	651 (173–2270)	673 (187–2326)	524 (146–1680)	<0.001	0.15
NT‐proBNP, pg/mL	1001 (275–3169)	957 (261–3182)	710 (204–2252)	<0.001	0.16
HbA_1c_, %	8.3 (6.9–10.8)	8.3 (6.9–10.8)	8.5 (7.1–11.0)	0.258	0.09
β blockers, n (%)	223 208 (90.0)	25 176 (90.1)	25 176 (90.1)	1.000	0
Renin–angiotensin system blockers (either ACE inhibitor or angiotensin receptor blocker or sacubitril/valsartan), n (%)	156 531 (63.1)	17 583 (62.9)	17 583 (62.9)	1.000	0
Mineralocorticoid receptor antagonists, n (%)	113 443 (45.7)	11 961 (42.8)	11 961 (42.8)	1.000	0
Implantable cardioverter defibrillators, n (%)	3683 (1.5)	438 (1.6)	339 (1.2)	<0.001	0.03
Cardiac resynchronization therapy, n (%)	1586 (0.6)	170 (0.6)	116 (0.4)	<0.001	0.03

ACE indicates angiotensin‐converting enzyme; BNP, brain‐type natriuretic peptide; eGFR, estimated glomerular filtration rate; HbA_1c_, hemoglobin A_1c_; HF, heart failure; NT‐proBNP, N‐terminal pro‐brain‐type natriuretic peptide; and SGLT‐2i, sodium‐glucose cotransporter‐2 inhibitor.

* The differences in the baseline characteristics between the patients using SGLT‐2is after the index date of HF diagnosis concerning proportion of days covered values of <80% and ≥80% are presented before propensity score matching analysis in Table S2.

**Figure 2:**
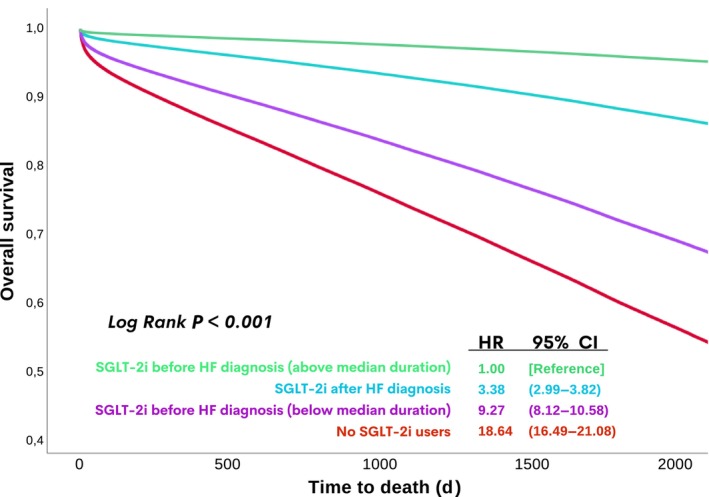
Comparison of SGLT‐2i users before index HF diagnosis vs after index HF diagnosis vs nonusers. HF indicates heart failure; HR, hazard ratio; and SGLT‐2i, sodium‐glucose cotransporter‐2 inhibitor.

First, the data revealed a significantly higher all‐cause mortality rate among patients with HF and diabetes who did not use an SGLT‐2i compared with those who were prescribed an SGLT‐2i, both before and after the index date of HF diagnosis (Figure 2). Notably, among patients with HF and diabetes, those who were prescribed an SGLT‐2i before HF diagnosis and who had a duration of exposure above the median duration had the lowest all‐cause mortality rate. This mortality rate was more favorable compared with patients with HF and diabetes who were prescribed an SGLT‐2i after the index date of HF diagnosis (Figure 2).

**Figure 3:**
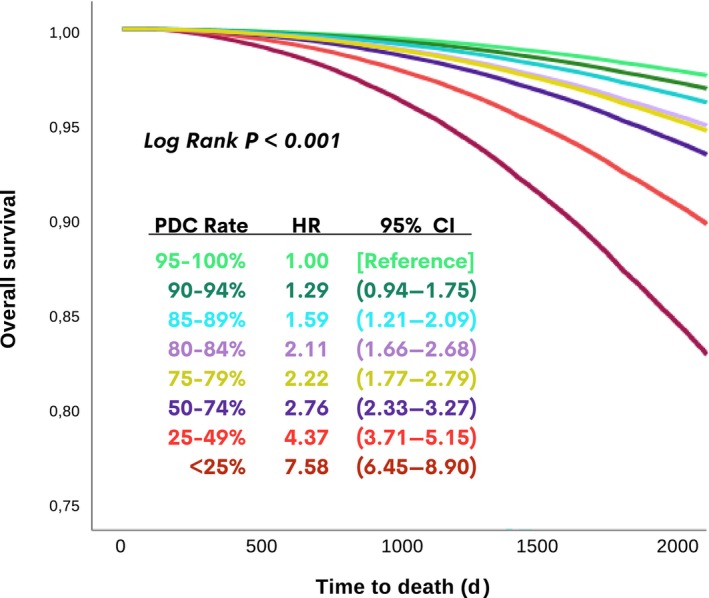
Comparison of SGLT‐2i users after the index diagnosis of HF according to subcategories of PDC adherence. HR indicates hazard ratio; and PDC, proportion of days covered.

Among patients with HF and diabetes who were prescribed an SGLT‐2i after the index HF diagnosis (n=213 113), median adherence was 21.4%, and median exposure to the SGLT‐2i was 306 days. Additionally, 13.1% (n=28 021) of SGLT‐2i users after the index diagnosis of HF had a PDC ≥80% (Figure 1). Among those patients who had an index diagnosis of HF after the year 2020 (ie, after publication of the first trial of SGLT‐2is in HF), the median adherence to the SGLT‐2i was found to be 46.3%. Of note, 32.9% and 22.1% of patients with HF and diabetes had an SGLT‐2i–related PDC ≥80% and ≥90%, respectively. In conformity with this, starting from a PDC <90%, all‐cause mortality risk significantly and gradually increased (hazard ratio [HR], 1.59 [95% CI, 1.21–2.09] for PDC, 85%–89%; Figure 3).

**Figure 4:**
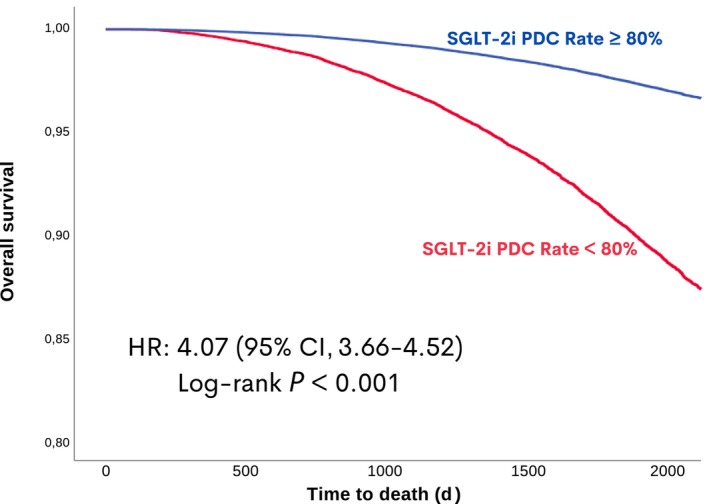
Comparison of SGLT‐2i users after the index diagnosis of HF in the propensity matched cohort for PDC <80% vs ≥80% adherence rate. HR indicates hazard ratio; PDC, proportion of days covered; and SGLT‐2i, sodium‐glucose cotransporter‐2 inhibitor.

Propensity score matching was then generated to compare patients with and without a PDC >80% adherence rate for SGLT‐2i after index date of HF diagnosis (Table). It was shown that, in the propensity score matching cohort including well matching for triple disease‐modifying therapies, patients having a PDC <80% were associated with a 4.07 times (HR [95% CI, 3.66–4.52]) increased all‐cause mortality risk compared with those with a PDC ≥80% (Figure 4).

Use of SGLT‐2is was associated with absolute risk reduction of 5.1% in total hospitalizations compared with nonusers of SGLT‐2is during follow‐up. Median hospitalization days were as follows: 5 days (interquartile range, 2–11) in SGLT‐2i users before the index date of HF diagnosis with above median duration, 6 days (interquartile range, 2–13) in SGLT‐2i users after the index date of HF diagnosis, and 7 days (interquartile range, 2–15) in nonusers of SGLT‐2is, respectively.

## Discussion

An exclusive class of antidiabetic medications known as SGLT‐2is prevents the proximal tubule from absorbing glucose and leads to glycosuria and better glycemic control.^14^ Cardiovascular outcome trials have shown that SGLT‐2is improve cardiovascular and renal outcomes, including reduced cardiovascular deaths and fewer hospitalizations for HF.^15^, ^16^ Hence, SGLT‐2is are effective in the treatment of type 2 diabetes and can be used in monotherapy or combination therapy. On the other hand, HF is a leading cause of death and morbidity worldwide, accounting for significant health costs, and type 2 diabetes is a frequent comorbidity in HF.^17^ Since the use of SGLT‐2is in patients with HF with or without type 2 diabetes have shown promising results, and contemporary guidelines recommend the addition of 2 SGLT‐2is, namely, dapagliflozin and empagliflozin, to standard treatment regimens for patients withHF with reduced ejection fraction and nonreduced ejection fraction to improve clinical outcomes.^18^, ^19^, ^20^, ^21^, ^22^ However, usage rates remain low in real life, particularly among the group of patients who can derive the most benefit.^23^

Adherence to SGLT‐2is could potentially be important not only for diabetic control but also for HF as well. Adherence to SGLT‐2is among patients with diabetes in a real‐life setting has been evaluated in a meta‐analysis. It was found that adherence to SGLT‐2is in a real‐life setting is poor, and a pooled proportion of people adherent to SGLT‐2is (PDC >80%) was found to be 49.0% at 1 year.^7^ Regarding HF, adherence to SGLT‐2is has been evaluated in a Canadian cohort of patients after an acute worsening of HF. After an acute worsening, among those who were prescribed an SGLT‐2i, adherence was >80% at 1 year.^8^ In this analysis, median adherence after the index HF diagnosis was overall low (21.4%) in a large cohort of patients with HF and diabetes. However, this might be related to reimbursement policy and prescription regulations in different countries. In Turkey, until April 2021, none of the SGLT‐2is was licensed as an HF medication, and hence cardiologists were not allowed to prescribe SGLT‐2is as an HF medication, requiring a consultation with an endocrinologist or an internist not only for prescription but also for reimbursement if the patient had diabetes. Although they have been licensed since then, they remain as out‐of‐pocket options unless the patient with HF has a concomitant diagnosis of diabetes. Therefore, overall good adherence, defined as PDC ≥80% was <13.1% in patients with HF and diabetes who were prescribed an SGLT‐2i after the index diagnosis of HF. Of note, median adherence increased from 21.4% to 46.3% among those who had an index diagnosis of HF after the year 2020.

In the pooled analysis of empagliflozin HF trials, it was shown that withdrawal of empagliflozin was linked to increased risk for cardiovascular death or HF hospitalization after 30 days of withdrawal.^6^ It was interesting to note that our real‐life data provided a somewhat confirmatory result regarding the prognostic importance of adherence to an SGLT‐2i, and in this cohort, an ≈10% decrease in PDC, which was almost equal to 30 days (based on the assumption of an overall median 306 days of exposure to the SGLT‐2i), was linked to a statistically increased risk for all‐cause death as well. Of note, a PDC with <80% adherence has usually been regarded as a standard definition for nonadherence^24^; however, regarding SGLT‐2i therapy, starting from <90% adherence, the risk for all‐cause death seemed to be increased.

To the best of our knowledge, there is neither a similar study in the literature that considers SGLT‐2i prescription timing relative to the index diagnosis of HF nor a study checking SGLT‐2i adherence with regard to prognosis.

## Limitations

There are some limitations worth mentioning. First, the distribution of patients with HF with reduced ejection fraction versus patients with non–HF with reduced ejection fraction is currently unknown since ejection fraction data were not reported separately in the database and *ICD* codes do not explicitly identify HF phenotype. We assume that the HF with reduced ejection fraction phenotype was the predominant phenotype at the time of index diagnosis, especially for those on quadruple therapy, but since both empagliflozin and dapagliflozin are recently approved HF medications, regardless of ejection fraction, HF phenotype is of less importance considering to adherence to these agents.^25^ Of note, echocardiographic data were available in the database; however, it was not technically possible to go over all the recorded images in such a big database. Second, since canagliflozin and other SGLT‐2is were not available in the country, findings regarding adherence and timing cannot be generalized. Throughout the bulk of the follow‐up period, both dapagliflozin and empagliflozin were mostly used as antidiabetic drugs to reduce high cardiovascular risk, with the evidence primarily obtained from EMPAREG‐OUTCOME (Empagliflozin Cardiovascular Outcome Event Trial in Type 2 Diabetes Mellitus Patients) trial data^15^ and partly from DECLARE (Dapagliflozin Effect on Cardiovascular Events) trial data.^16^ Since the DAPA‐HF (Dapagliflozin and Prevention of Adverse Outcomes in Heart Failure)^26^ and EMPEROR‐REDUCED (Empagliflozin Outcome Trial in Patients With Chronic Heart Failure and a Reduced Ejection Fraction) trials^27^ appeared later, glucometabolic control was the primary focus at earlier times. Hence, the observed adherence rate was found to be higher in the years after the publication of these major papers.

In the cohort, adherence to SGLT‐2is was solely considered in‐depth via prescription data, not other agents. However, it is well known that patients who are nonadherent to 1 class of agents are usually those who are nonadherent to many other agents. Hence, the association of lower adherence to SGLT‐2is and increased risk of all‐cause death might be magnified by lower exposure to other disease‐modifying medications in the cohort before the index HF diagnosis. Of note, in the propensity cohort matched group, after index HF diagnosis, SGLT‐2i adherence was still significantly important among patients with HF and diabetes.

The COVID‐19 pandemic may influence hospitalization data, as observed globally.^28^ In light of this, it is crucial to interpret the hospitalization results from our study with an understanding of the potential confounding effects of COVID‐19. Although the study excluded hospitalization records for confirmed COVID‐19 cases, the influence of the pandemic on overall hospitalization rates may have partially affected our data. Detailed analyses addressing this potential impact were not conducted. It is important to note that our analysis encompassed all‐cause hospitalizations rather than those specifically attributable to HF.

Moreover, since diabetes data were gathered only as a comorbidity, we were unable to determine the duration of diabetes in this sample. Therefore, it is still unclear if the duration of diabetes matters in this context. Furthermore, the observed differences cannot be applied to the entire spectrum of HF because they do not represent individuals with HF without type 2 diabetes.

In conclusion, out of a nationwide cohort, SGLT‐2is as a class generate robust all‐cause death benefit among patients with HF and diabetes, and the benefit was stronger if they were prescribed before the index diagnosis of stage C HF along with good enough exposure time (>417 days, ie, exceeding 1 year) designating the importance of early initiation and continuation. On the other hand, decreased adherence to SGLT‐2is after the index diagnosis of HF resulted in increased all‐cause mortality risk, which became statistically significant when there was <90% PDC adherence to SGLT‐2is.

## Sources of Funding

None.

## Disclosures

MBY reports institutional fee from Novartis, Bayer, Amgen, Novo Nordisk, Astra Zeneca, Boehringer Ingelheim, Albert Health and support for attending meetings from Menarini. All other authors report None.

## Supporting information

Tables S1–S2
