# Supplementary material for: Timing and Adherence Matter for Sodium–Glucose Cotransporter‐2 Inhibitors in Heart Failure
Source: J Am Heart Assoc. 2025 Mar 21;14(7):e037035. doi: 10.1161/JAHA.124.037035 (PMC12132807; doi:10.1161/JAHA.124.037035)
Supplement: Supplementary file 1 — Tables S1–S2 [file JAH3-14-e037035-s001.pdf]

SUPPLEMENTAL MATERIAL

Table S1: Definitions for heart failure, diabetes mellitus, comorbidities and drugs

**Heart failure**	ICD I50.0 (Congestive heart failure)<br><br>ICD I50.1 (Left ventricular dysfunction)<br><br>ICD I50.9 (HF, unspecified)<br><br>ICD I11.0 (Hypertensive heart disease with congestive heart failure)<br><br>ICD I13.0 (Hypertensive heart and chronic kidney disease with congestive heart failure)<br><br>ICD I13.1<br><br>ICD I13.2 (Hypertensive heart and chronic kidney disease with congestive heart failure and renal failure)<br><br>ICD I13.9<br><br>ICD I42.0 (Dilated cardiomyopathy).<br><br>AND<br><br>• Prescription of at least one major heart failure medication, i.e., angiotensin converting enzyme inhibitors, angiotensin receptor blockers, angiotensin receptor blocker-neprilysin inhibitor, beta-blockers, mineralocorticoid receptor blockers, sodium glucose co-transporter 2 inhibitors, and loop diuretics
**Diabetes mellitus**	E10 (Insulin-dependent diabetes mellitus)<br><br>E11 (Insulin nondependent diabetes mellitus)<br><br>E13, E14 (Unspecified diabetes mellitus)
**Chronic Obstructive Pulmonary Disease**	J44 (Chronic obstructive pulmonary disease, other)
**Atrial fibrillation**	I48 (Atrial fibrillation and flutter)
**Anemia**	D63 (Anemia, in chronic diseases classified elsewhere)<br><br>D64 (Anemia, other)
**Prior myocardial infarction**	I21(Acute myocardial infarction)
**Drugs ATC Codes**	
**Beta-blockers**	C07AB
**RAAS inhibitors**	C09
**MRA**	C03D

Table S2: Baseline characteristics of the SGLT2i users after heart failure index diagnosis and the differences between the two groups regarding adherence rate before propensity score matching analysis.

Characteristics	All patients on SGLT2i after HF index diagnosis (n=213,113)	<80% adherence rate (n=185,092)	≥80% adherence rate (n=28,021)	P value
Age, mean ± SD, years	63.5 ± 9.8	63.6 ± 9.8	62.5 ± 9.5	<0.001
Sex (Female)	108,994 (51.1)	94,999 (51.3)	13,995 (49.9)	<0.001
Anemia, n (%)	96,163 (45.1)	83,869 (45.3)	12,294 (43.9)	<0.001
Chronic obstructive pulmonary disease, n (%)	93,254 (43.8)	82,548 (44.6)	10,706 (38.2)	<0.001
History of myocardial infarction, n (%)	57,481 (27.0)	49,808 (26.9)	7673 (27.4)	0.096
Atrial fibrillation, n (%)	78,138 (36.7)	69,634 (37.6)	8504 (30.3)	<0.001
eGFR, mL/min/1.73 m2	81.6 (63.3-95.3)	81.0 (62.8-94.9)	84.3 (66.0-96.9)	<0.001
Potassium, mmol/L	4.2 (3.8-4.6)	4.3 (3.9-4.6)	4.2 (3.8-4.6)	0.317
Uric acid, mg/dL	5.5 (4.4-6.9)	5.6 (4.4-7.0)	5.2 (4.2-6.4)	0.697
Hemoglobin, g/dL	12.9 (10.0-15.6)	12.8 (10.0-15.6)	13.2 (10.2-16.0)	<0.001
BNP, pg/ml	680 (183-2348)	705 (189-2456)	524 (146-1679)	<0.001
NT-proBNP, pg/ml	1053 (292-3311)	1116 (308-3508)	710 (204-2251)	<0.001
HbA1c, %	8.2 (6.9-10.8)			0.347
Beta-blockers, n (%)	192,569 (90.4)	167,362 (90.4)	25,207 (90.0)	0.014
Renin angiotensin system blockers (either ACE inhibitor or angiotensin receptor blocker or sacubitril/valsartan), n (%)	135,615 (63.6)	118,007 (63.8)	17,608 (62.8)	0.003
Mineralocorticoid receptor antagonists, n (%)	101,315 (47.5)	89,303 (48.2)	12,012 (42.9)	<0.001
Implantable cardioverter defibrillators, n (%)	3501 (1.6)	3161 (1.7)	340 (1.2)	<0.001
Cardiac resynchronization therapy, n (%)	1534 (0.7)	1418 (0.8)	116 (0.4)	<0.001
